# Bintrafusp Alfa, a Bifunctional Fusion Protein Targeting TGF-β and PD-L1, in Patients with Non-Small Cell Lung Cancer Resistant or Refractory to Immune Checkpoint Inhibitors

**DOI:** 10.1093/oncolo/oyac253

**Published:** 2022-12-26

**Authors:** Fabrice Barlesi, Nicolas Isambert, Enriqueta Felip, Byoung Chul Cho, Dae Ho Lee, Julio Peguero, Guy Jerusalem, Nicolas Penel, Esma Saada-Bouzid, Pilar Garrido, Christoph Helwig, George Locke, Laureen S Ojalvo, James L Gulley

**Affiliations:** Department of Medical Oncology, Gustave Roussy Cancer Campus, Villejuif, France; Service d’oncologie médicale CLCC Georges-François Leclerc, Dijon, France; Oncology Department, Vall d’Hebron University Hospital and Institute of Oncology (VHIO), UVic-UCC, IOB-Quiron, Barcelona, Spain; Yonsei Cancer Center, Yonsei University College of Medicine, Seoul, Republic of Korea; Department of Oncology, University of Ulsan College of Medicine, Seoul, Republic of Korea; Department of Research, Oncology Consultants, Houston, TX, USA; Medical Oncology, CHU Sart Tilman Liege and Liege University, Domaine Universitaire, Liege, Belgium; Department of Medical Oncology, Lille University, Medical School and Centre Oscar Lambret, Lille, France; Department of Medical Oncology, Early Phase Trials Unit, Centre Antoine Lacassagne, Nice, France; Lung Cancer Unit, University Hospital Ramón y Cajal (IRYCIS), Medical Oncology Department, Madrid, Spain; Merck Healthcare KGaA, Darmstadt, Germany; EMD Serono Research & Development Institute, Inc, Billerica, MA, USA (an affiliate of Merck KGaA); EMD Serono Research & Development Institute, Inc, Billerica, MA, USA (an affiliate of Merck KGaA); Genitourinary Malignancies Branch, National Cancer Institute, National Institutes of Health, Bethesda, MD, USA

**Keywords:** Bintrafusp alfa, TGF-β, PD-L1, bifunctional, non-small cell lung cancer (NSCLC)

## Abstract

**Background:**

Bintrafusp alfa is a first-in-class bifunctional fusion protein composed of the extracellular domain of transforming growth factor beta receptor II (a TGF-β “trap”) fused to a human immunoglobulin G1 monoclonal antibody blocking programmed cell death 1 ligand 1 (PD-L1). We report the efficacy and safety in patients with non-small cell lung cancer (NSCLC) that progressed following anti-PD-(L)1 therapy.

**Materials and Methods:**

In this expansion cohort of NCT02517398—a global, open-label, phase I trial—adults with advanced NSCLC that progressed following chemotherapy and was primary refractory or had acquired resistance to anti-PD-(L)1 treatment received intravenous bintrafusp alfa 1200 mg every 2 weeks until confirmed progression, unacceptable toxicity, or trial withdrawal. The primary endpoint was best overall response (by Response Evaluation Criteria in Solid Tumors version 1.1 adjudicated by independent review committee); secondary endpoints included safety.

**Results:**

Eighty-three eligible patients (62 [74.7%] treated with ≥3 prior therapies) received bintrafusp alfa. Four patients (3 primary refractory, 1 acquired resistant) had confirmed partial responses (objective response rate, 4.8%; 95% CI, 1.3%-11.9%), and 9 had stable disease. Tumor cell PD-L1 expression was not associated with response. Nineteen patients (22.9%) experienced grade ≥3 treatment-related adverse events, most commonly asthenia (3 [3.6%]) and fatigue, eczema, and pruritus (2 each [2.4%]). One patient had grade 4 amylase increased. One patient died during treatment for pneumonia before initiation of bintrafusp alfa.

**Conclusion:**

Although the primary endpoint was not met, bintrafusp alfa showed some clinical activity and a manageable safety profile in patients with heavily pretreated NSCLC, including prior anti-PD-(L)1 therapy. Tumor responses occurred irrespective of whether disease was primary refractory or had acquired resistance to prior anti-PD-(L)1 therapy.

## Teaser

This article reports clinical activity and manageable safety from an expansion cohort of patients with advanced non-small cell lung cancer that was primary refractory or had acquired resistance to anti-PD-(L)1 therapy from a phase I study of bintrafusp alfa.

**Implications for Practice**

No established standard of care exists for patients with advanced NSCLC and disease progression following anti-PD-(L)1 therapy. Increased expression of TGF-β is associated with poor response and reduced survival in some cancers. This article reports clinical activity and manageable safety from an expansion cohort of patients with advanced NSCLC that was primary refractory or had acquired resistance to anti-PD-(L)1 therapy from a phase I study (NCT02517398) of bintrafusp alfa, an experimental first-in-class bifunctional fusion protein composed of the extracellular domain of TGF-βRII (a TGF-β “trap”) fused to a human IgG1 mAb blocking PD-L1. Further investigation of bintrafusp alfa in solid tumors as monotherapy or in combination with other agents is ongoing.

## Introduction

Non-small cell lung cancer (NSCLC) is the most common type of lung cancer,^1^ and approximately 80% of patients with NSCLC have advanced disease at diagnosis.^2^ Before the introduction of immune checkpoint inhibitors, fewer than 5% of patients with metastatic NSCLC survived longer than 5 years.^2^

Emerging evidence indicates that the tumor microenvironment (TME) plays an important role in the pathology of NSCLC.^3^ An important factor in the TME is transforming growth factor beta (TGF-β) signaling, which can promote tumor immune evasion and tumor progression via both the innate and adaptive immune systems—primarily through suppression of tumor immune surveillance by cytotoxic T, natural killer, and dendritic cells.^4-6^ In addition, TGF-β inhibits tumor-suppressing macrophage and neutrophil development but promotes the activation of tumor-promoting macrophages and neutrophils and regulatory T-cell differentiation, and mediates the function of myeloid-derived suppressor cells.^7,8^ TGF-β affects additional processes relevant to tumor pathogenesis, such as epithelial-to-mesenchymal transition (EMT), fibrosis, and tumor angiogenesis.^4,5^ Notably, TGF-β activity has been implicated in promoting NSCLC progression, metastasis, and drug resistance.^9^

Another important signaling pathway in the TME is the programmed cell death 1 ligand 1 (PD-L1) pathway, as demonstrated by the efficacy of anti-programmed cell death 1 protein (PD-1) or anti-PD-L1 therapies for NSCLC.^10^ Anti-PD-1/PD-L1 (anti-PD-(L)1) therapies have demonstrated efficacy as first-line (1L) and later treatments in patients naïve to anti-PD-(L)1 with advanced NSCLC. Objective response rates (ORRs) vary for NSCLC 1L anti-PD-(L)1 monotherapies, ranging from 12% to 20% for patients treated without PD-(L)1 selection criteria and 14% to 44.8% for patients with PD-L1-positive tumors.^11-17^ ORRs for anti-PD-(L)1 therapies in 1L combination regimens, which included chemotherapy, cytotoxic T-lymphocyte antigen-4 inhibitors, and vascular endothelial growth factor-specific angiogenesis inhibitors, ranged from 36% to 64% for 1L combination regimens.^17-21^ No established standard of care exists for patients with advanced, pretreated NSCLC who have had disease progression following anti-PD-(L)1 therapy,^22^ highlighting the need for novel treatment options for these patients.

Increased expression of TGF-β is associated with a lack of response to PD-L1 blockade and reduced survival in some cancers,^23^ and inhibition of the TGF-β pathway may help patients with NSCLC to overcome resistance to anti-PD-(L)1 treatment. Reduced TGF-β signaling in stromal cells, antitumor immunity and regression, and facilitated T-cell penetration into the center of the tumor have been found in preclinical studies investigating the combination of a TGF-β inhibitor and anti-PD-L1 agent.^23-25^

Bintrafusp alfa is a first-in-class bifunctional fusion protein composed of the extracellular domain of the human TGF-β receptor II (TGF-βRII or TGF-β “trap”) fused via a flexible linker to the C terminus of each heavy chain of an IgG1 antibody blocking anti-PD-L1), which might allow for colocalized, simultaneous inhibition of TGF-β and PD-L1 in the TME.^25,26^ In preclinical models, bintrafusp alfa resulted in superior tumor regression compared with either an anti-PD-L1 antibody or a “trap” control.^25^ Bintrafusp alfa also reversed EMT in human lung cancer cell lines.^27^

The phase I trial NCT02517398 is investigating the safety and efficacy of bintrafusp alfa in patients with advanced/pretreated solid tumors and includes multiple expansion cohorts with specific tumor types. In the dose-escalation cohort of this study, bintrafusp alfa had a manageable safety profile and demonstrated clinical activity in patients with heavily pretreated advanced solid tumors.^26^ Bintrafusp alfa also demonstrated clinical activity as second-line (2L) treatment in an NSCLC expansion cohort.^28^ Here, we describe the clinical outcomes of bintrafusp alfa treatment in patients with stage IV metastatic or recurrent NSCLC that was primary refractory (defined as disease that did not respond or progressed as the best overall response following treatment initiation) or had acquired resistance (defined as initial disease control with subsequent disease progression) to prior treatment with anti-PD-(L)1 therapy.

## Materials and Methods

### Study Design and Patients

This expansion cohort from the global, multicenter, phase I, open-label NCT02517398 study of bintrafusp alfa evaluated patients with advanced NSCLC that was primary refractory (ie, best overall response of progressive disease on prior anti-PD-(L)1 therapy) or had acquired resistance to anti-PD-(L)1 therapy (ie, best overall response of complete/partial response or stable disease, with subsequent progressive disease on prior anti-PD-(L)1 therapy).

Eligible patients were ≥18 years of age, with histologically confirmed stage IV (metastatic) or recurrent NSCLC (per 7th International Association for the Study of Lung Cancer classification). Disease had to be measurable by Response Evaluation Criteria in Solid Tumors version 1.1 (RECIST 1.1). Fresh biopsies taken within 28 days before the first study treatment administration had to be available. At a minimum, patients had to have had disease progression following anti-PD-(L)1 as monotherapy and also following platinum-based chemotherapy, and they should have exhausted other appropriate standard treatment options. Anti-PD-(L)1 monotherapy was not required to be the most recent prior treatment. In addition, patients had to have an Eastern Cooperative Oncology Group performance status of 0 or 1, life expectancy of ≥12 weeks, and adequate renal, hepatic, and hematologic function. Patient selection was not based on PD-L1 expression or other biomarkers.

The study was conducted in accordance with all applicable regulatory requirements, and the protocol was approved by the institutional review board of the participating institutions. All patients provided written informed consent before study enrollment. The study complied with international standards of Good Clinical Practice and the Declaration of Helsinki.

### Procedures

Patients received bintrafusp alfa 1200 mg once every 2 weeks (Q2W) via intravenous infusion over 1 h until confirmed progressive disease, unacceptable toxicity, or trial withdrawal; treatment past disease progression was permitted if clinically justified. This regimen was chosen for all expansion cohorts based on integrated analysis of bintrafusp alfa exposure, response, and progression-free survival (PFS).^29^ To mitigate potential infusion-related reactions, premedication with an antihistamine and acetaminophen given 30-60 min before each dose of bintrafusp alfa was mandatory for at least the first 2 infusions. Steroids were not permitted as premedication. Interruption or discontinuation of bintrafusp alfa was allowed if treatment-related adverse events (TRAEs), infusion-related reactions of grade ≥2 severity, or severe or life-threatening adverse events occurred. However, dose reduction was not permitted according to the trial protocol.

Tumor response was assessed by radiographic imaging 6 weeks after starting treatment, then every 6 weeks for the first year and every 12 weeks thereafter. Response was confirmed by repeated radiographic assessment 4 weeks or longer from the first documented response. To evaluate the safety of bintrafusp alfa, adverse events were monitored throughout treatment: at 28 days after the last study dose, at 10 weeks post treatment, and every 12 weeks thereafter.

### Biomarker Analysis

Biomarker analysis was performed as previously described.^28^ Tumor PD-L1 protein expression was obtained from fresh biopsy specimens at baseline and measured by immunohistochemical staining of formalin-fixed, paraffin-embedded blocks with a proprietary assay (Dako, Carpinteria, CA) using anti-PD-L1 monoclonal antibody clone 73-10. PD-L1 positivity in tumors was scored as the proportion of tumor cells showing membranous PD-L1 staining; PD-L1-positive disease was defined as ≥1% of tumor cells showing detectable PD-L1 expression. Analysis of TGF-β1 concentrations in the TME after treatment was not conducted. Gene expression of pretreatment archival formalin-fixed, paraffin-embedded tumor samples was analyzed using RNAseq. Sequencing reads were aligned against the hg19 reference genome using Bowtie2 v2.2.3. Gene expression was determined using RSEM v1.2 with Ensembl gene annotations. Transcript-per-million (TPM) values were upper-quintile normalized for further analysis. After quality control, gene expression was quantified in samples as log-TPM, given by log_2_(0.5+TPM). Differential gene expression between patients with nonprogressive disease (response or stable disease) vs progressive disease was evaluated using DESeq2 v1.14.1 in R v3.32.

The tumor mutation count was measured with an RNAseq-based variant calling that used tumor RNAseq data combined with germline, normal whole-exome sequencing to produce a set of tumor-specific mutations. Tumor samples were sequenced at 2 × 50 to a target of 10^8^ read pairs with an Illumina HiSeq System (Illumina, San Diego, CA). Whole-exome sequencing was performed by Expression Analysis (Research Triangle Park, NC) using matched peripheral blood samples and an Agilent SureSelect Human All Exon V5 kit (Agilent Technologies, Santa Clara, CA). Sequencing was done on an Illumina HiSeq System with a target of 100 × coverage. Sequencing reads were mapped to hg19 and the Ensembl gene annotations (ensGene; University of California, Santa Cruz, CA) using RNA-STAR v2.5.0b; whole-exome reads were mapped to hg19 using BWA-MEM v0.7.12.^30,31^ Mutation calling was performed on paired BAM files using VarDictJava v1.4.2.^32^ Results were annotated using Ensembl Variant Effect Predictor version 85 to determine the location and type of mutation.^33^ We define “tumor mutation count” for a given patient as the total count of all missense mutations discovered for that patient.

### Outcomes

The primary endpoint was the best overall response assessed according to RECIST 1.1 and assessed by the independent review committee (IRC). Secondary endpoints included the best overall response per investigator and safety, with adverse events coded according to Medical Dictionary for Regulatory Activities terms version 21.0 and classified by grade according to the National Cancer Institute Common Terminology Criteria for Adverse Events version 4.03. Exploratory endpoints included duration of response, PFS, and time to progression (all determined per RECIST 1.1 by the IRC), and overall survival (OS).

### Statistical Analyses

This expansion cohort was planned to enroll 80 patients. With 80 patients treated, the study has approximately 92% and 99% power to rule out a ≤5% ORR (null hypothesis) when the true ORRs are 15% and 20%, respectively, at the 5% type I error rate (1 sided). Efficacy and safety were analyzed in all patients who received ≥1 dose of bintrafusp alfa. The ORR was determined as the proportion of patients with a confirmed best overall response of complete response (CR) or partial response (PR). The uncertainty of estimates was assessed by calculating a 95% exact (Clopper-Pearson) CI. The disease control rate (DCR) was defined as the proportion of patients with a confirmed best overall response of CR, PR, stable disease, or non-CR/nonprogressive disease. The duration of response was analyzed using the Kaplan-Meier method, as were PFS, time to progression, and OS. Safety was analyzed using descriptive statistics.

## Results

Between September 1, 2016, and March 8, 2017, 132 patients with advanced, pretreated NSCLC who had disease progression following anti-PD-(L)1 as monotherapy were screened for enrollment. The 83 patients from 32 study sites in North America, Europe, and the Asia-Pacific region who met the criteria for eligibility were enrolled in this expansion cohort and were included in the full analysis and safety sets (Supplementary Table SA.1). Of the 49 patients not enrolled, 44 did not meet the eligibility criteria, 2 withdrew consent, 1 had a stroke and was hospitalized, 1 had an adverse event, and 1 died.

This population was heavily pretreated, with 62 of 83 patients (74.7%) having received ≥3 prior anticancer regimens and 43 (51.8%) having received anti-PD-(L)1 therapy as their most recent treatment (Table 1). The majority of patients were male (67.5%), and the median age was 63 years (range, 25-88 years); 46 patients (55.4%) were aged <65 years. Two-thirds of patients (66.3%) had nonsquamous primary histology; 35 patients (42.2%) had primary refractory disease (ie, no disease control with prior anti-PD-(L)1 therapy), 45 (54.2%) had acquired resistance (ie, disease control with prior anti-PD-(L)1 therapy, followed by subsequent progression), and prior PD-(L)1 treatment status was not available for 3 patients (3.6%). Four patients (7.3% of patients with nonsquamous histology) had tumors with an *EGFR* mutation (Table 1), and no patients had tumors with an *ALK* translocation or *ROS1* rearrangement. All 4 patients with *EGFR* mutations had received previous EGFR inhibitor therapy.

**Table 1.** Baseline patient and disease characteristics (*N* = 83).

Characteristic	Values^*^
Sex	
Male	56 (67.5)
Female	27 (32.5)
Age	
Median (range), years	63 (25-88)
<65 years	46 (55.4)
≥65 years	37 (44.6)
ECOG performance status	
0	27 (32.5)
1	55 (66.3)
2	1 (1.2)
Tumor cell PD-L1 expression^a^	
≥1%	54 (65.1)
<1%	21 (25.3)
Not available	8 (9.6)
*EGFR* mutation status^b^	
Wild type	47 (85.5)
Mutated	4 (7.3)
Not available	4 (7.3)
Tumor histology	
Nonsquamous^c^	55 (66.3)
Squamous cell carcinoma	28 (33.7)
Number of prior anticancer regimens^d^	
2	21 (25.3)
3	26 (31.3)
≥4	36 (43.4)
Type of prior anticancer therapy for metastatic or locally advanced disease	
Anti-PD-(L)1	83 (100)
Cytotoxic therapy	79 (95.2)
Endocrine therapy	1 (1.2)
Monoclonal antibody therapy	19 (22.9)
Small molecules	18 (21.7)
Immunotherapy other than anti-PD-(L)1	3 (3.6)
Other	7 (8.4)
Last anticancer therapy before the study	
Anti-PD-(L)1	43 (51.8)
Cytotoxic therapy	36 (43.3)
Monoclonal antibody therapy	4 (4.8)
Small molecules	5 (6.0)
Immunotherapy other than anti-PD-(L)1	2 (2.4)
Other	1 (1.2)
Best objective response to last anticancer regimen	
Complete response	0
Partial response	10 (12.0)
Stable disease	30 (36.1)
Progressive disease	41 (49.4)
Not assessable	1 (1.2)
Not available	1 (1.2)
Prior PD-(L)1 treatment status	
Acquired resistance	45 (54.2)
Primary refractory	35 (42.2)
Not available	3 (3.6)

^*^Values are shown as *n* (%) unless otherwise indicated.

^a^PD-L1 expression was determined by immunohistochemistry using a proprietary assay (Dako PD-L1 IHC 73-10 pharmDx; Dako, Carpinteria, CA).^28^

^b^Percent was calculated based on the number of patients with nonsquamous histology (*n* = 55).

^c^Nonsquamous histology includes adenocarcinoma (*n* = 50), large cell (*n* = 1), non-small cell carcinoma (*n* = 1), epidermoid sarcoma (*n* = 1), epidermoid carcinoma (*n* = 1), and sarcomatoid carcinoma (*n* = 1).

^d^Prior therapy in any setting (neoadjuvant, adjuvant, locally advanced, and metastatic disease).

Abbreviations: ECOG, Eastern Cooperative Oncology Group; PD-L1, programmed cell death 1 ligand 1.

At the data cutoff of August 24, 2018, patients received bintrafusp alfa for a median duration of 8.0 weeks (range, 2.0-70.0 weeks). The median follow-up time by Kaplan-Meier analysis was 77.0 weeks (range, 0.3-92.7 weeks). At the data cutoff, no patients remained on treatment, but 15 (18.1%) were still in the study for follow-up. The most common reason for treatment termination was disease progression in 55 patients (66.3%). Thirty patients (36.1%) had subsequent anticancer drug therapy, with the majority (*n* = 22 [26.5%]) receiving subsequent cytotoxic therapy. Of the 68 patients (81.9%) who discontinued the study, 62 died (9 while on treatment), 4 withdrew consent, and 2 were lost to follow up.

**Figure 1.**
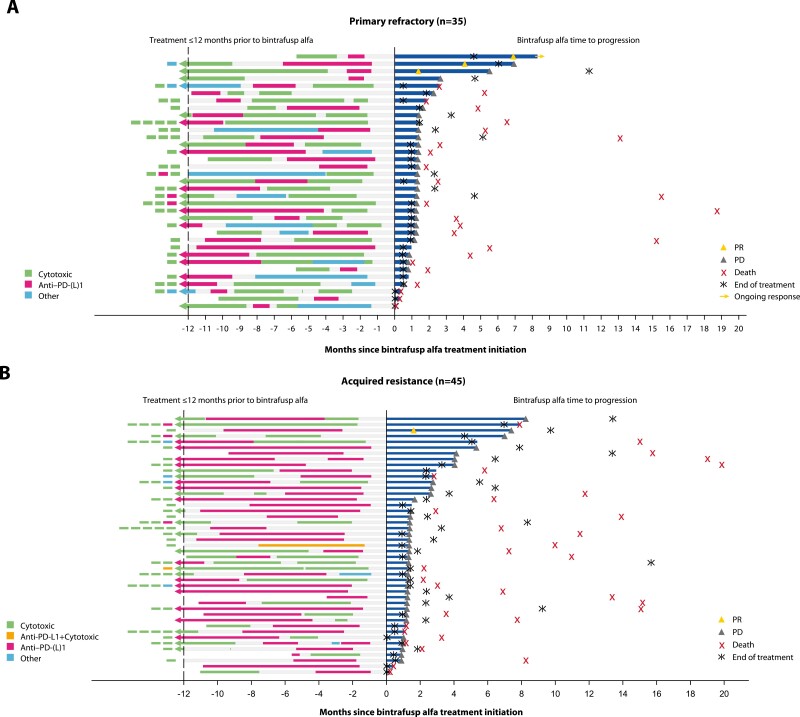
Prior treatment and time to progression on bintrafusp alfa treatment.^a^ Prior treatment and disease response to bintrafusp alfa in patients with non-small cell lung cancer that was primary refractory, ie, no disease control with prior anti-PD-(L)1 therapy (A) or had acquired resistance, ie, initial disease control with prior anti-PD-(L)1 therapy, followed by subsequent progression (B). ^a^Boxes left of the vertical dashed line indicate sequence of treatment beyond 12 months prior to first dose of bintrafusp alfa. Abbreviations: PD, progressive disease; PD-L1, programmed cell death 1 ligand 1; PR, partial response.

Four patients had a confirmed PR per IRC and per investigator (ORR, 4.8% [95% CI, 1.3%-11.9%] for both IRC and investigator assessment; Table 2 and Supplementary Table SA.2). At the data cutoff, the DCR per IRC was 18.1% (95% CI, 10.5%-28.1%); 4 patients had PRs, 9 had stable disease, and 2 had non-CR/nonprogressive disease. The DCR per the investigators’ assessments was 25.3% (95% CI, 16.4%-36.0%); 4 patients had PRs and 17 had stable disease (Supplementary Table SA.2). The median duration of response per IRC was 4.2 months (95% CI, 2.9-5.8 months); 1 patient had an ongoing response at data cutoff but had discontinued treatment due to an adverse event. All 4 patients had received anti-PD-(L)1 as their last therapy prior to bintrafusp alfa (Fig. 1).

**Table 2.** Summary of response data by IRC.

Response	Primary refractory (*n* = 35)^a^	Acquired resistance (*n* = 45)^a^	Total (*N* = 83)
Best overall response			
Complete response	0	0	0
Partial response	3 (8.6)	1 (2.2)	4 (4.8)
Stable disease	0	9 (20.0)	9 (10.8)
Non-complete response/nonprogressive disease^b^	0	2 (4.4)	2 (2.4)
Progressive disease	25 (71.4)	28 (62.2)	54 (65.1)
Not evaluable^c^	7 (20)	5 (11.1)	14 (16.9)
Objective response	3 (8.6; 1.8-23.1)	1 (2.2; 0.1-11.8)	4 (4.8; 1.3-11.9)
Disease control rate	3 (8.6; 1.8-23.1)	12 (26.7; 14.6-41.9)	15 (18.1; 10.5-28.1)

Data are *n* (%), *n* (%; 95% CI), or median (range or 95% CI), according to IRC-assessed Response Evaluation Criteria in Solid Tumors version 1.1.

^a^Primary refractory or acquired resistance status was not available for 3 of the 83 patients.

^b^Non-complete response/non-progressive disease was defined as the persistence of one or more non-target lesion(s) and/or maintenance of tumor marker level above the normal limits in non-target lesion.

^c^Of 14 patients, 9 (64.3%) had no tumor review by the IRC.

Abbreviation: IRC, independent review committee.

**Figure 2.**
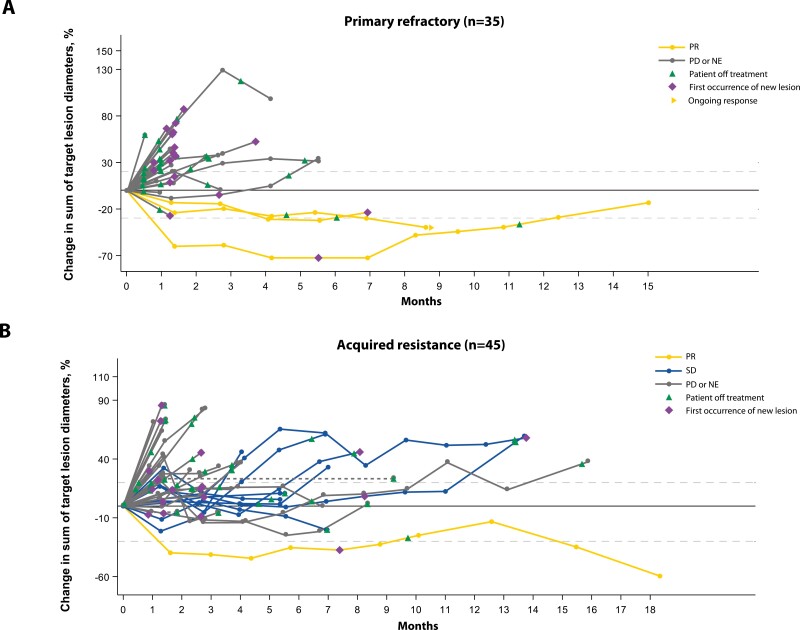
Clinical activity of bintrafusp alfa. Percentage change in target lesion diameters over time as adjudicated by the IRC per RECIST 1.1 in patients with NSCLC that was primary refractory, ie, no disease control with prior anti-PD-(L)1 therapy (A) or had acquired resistance, ie, initial disease control with prior anti-PD-(L)1 therapy, followed by subsequent progression (B). Dashed lines at 20% and –30% indicate thresholds for progressive disease and partial response, respectively. Abbreviations: IRC, independent review committee; NE, not evaluable; NSCLC, non-small cell lung cancer; PD, progressive disease; PR, partial response; RECIST 1.1, Response Evaluation Criteria in Solid Tumors version 1.1; SD, stable disease.

**Figure 3.**
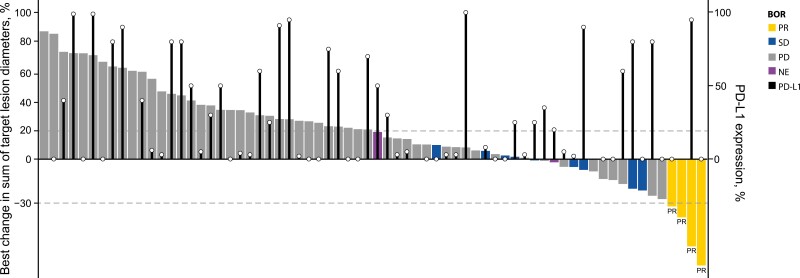
Best percentage change in target lesions from baseline as assessed by the IRC, according to PD-L1 expression level. ^a^Black bars indicate level of PD-L1 expression (%). Abbreviations: BOR, best overall response; IRC, independent review committee; NE, not evaluable; PD, progressive disease; PD-L1, programmed cell death 1 ligand 1; PR, partial response; SD, stable disease.

Clinical activity of bintrafusp alfa was noted in patients with a range of prior treatments and treatment responses and irrespective of tumor PD-L1 expression that was obtained from fresh baseline tumor tissue (Figs. 1-3 and Supplementary Fig. SA.1). Disease control per IRC was observed both in patients who had primary refractory disease and in those who developed acquired resistance to prior anti-PD-(L)1 therapy (Fig. 2 and Supplementary Fig. SA.1.A). Most IRC-assessed PRs were observed in patients with primary refractory disease, but more patients with acquired resistance had stable disease with bintrafusp alfa (Table 2; Fig. 2). Of the 4 patients who had a response, 2 had tumor PD-L1 expression <1%, 1 had tumor PD-L1 expression ≥1%, and 1 was not evaluable for PD-L1 expression.

In the 43 patients who received anti-PD-(L)1 as their most recent therapy prior to bintrafusp alfa, the confirmed ORR per IRC was 9.3% (95% CI, 2.6%-22.1%). Disease control was more commonly achieved in patients who had anti-PD-(L)1 vs. other therapy as their last therapy prior to bintrafusp alfa (DCR, 23.3% [95% CI, 11.8%-38.6%] vs. 12.5% [95% CI, 4.2%-26.8%], respectively) (Fig. 1 and Supplementary Fig. SA.1.B).

Overall, the IRC-assessed median PFS was 1.3 months (95% CI, 1.3-1.4 months; Supplementary Fig. SA.2). The 6-month PFS rate was 9.6% in the overall cohort, and 6.1% (95% CI, 1.1%-17.7%) and 13.2% (95% CI, 5.0%-25.3%) in the patients with primary refractory and acquired resistant disease, respectively. The median OS was 6.8 months (range, 0-21 months [95% CI, 4.4-11.5 months]; Supplementary Fig. SA.3), 4.8 months (range, 0-21 months [95% CI, 2.5-13.1 months]) in patients with primary refractory disease, and 10.0 months (range, 0-20 months [95% CI, 6.4-15.0 months]) in patients with acquired resistance. In the subgroup of patients who received anti-PD-(L)1 as their most recent therapy prior to bintrafusp alfa, the median PFS was 1.4 months (95% CI, 1.3-1.7 months), and the median OS was 7.3 months (95% CI, 4.8-13.1 months).

Sixty-one patients (73.5%) experienced TRAEs, of which the most common were asthenia (*n* = 23 [27.7%]), pruritus (*n* = 19 [22.9%]), and decreased appetite (*n* = 14 [16.9%]). Nineteen patients (22.9%) experienced TRAEs of grade ≥3, of which the most common were asthenia (*n* = 3 [3.6%]), pruritus (*n* = 2 [2.4%]), fatigue (*n* = 2 [2.4%]), and eczema (*n* = 2 [2.4%]) (Table 3). One patient had a grade 4 event (amylase increased). Another patient developed grade 3 pneumonia with parapneumonic pleural effusion after study entry and 9 days before the start of study treatment. The event worsened over the course of days, leading to death on day 8 after the first dose of bintrafusp alfa. The death was assessed as treatment-related death by the investigator. Four patients (4.8%) discontinued treatment because of a TRAE (acute kidney injury in 2 patients, adrenal insufficiency and eczema in 1 each). Immune-related adverse events occurred in 6 patients (7.2%), of which 2 (2.4%) were grade 3 (Supplementary Table SA.3). One patient (1.2%) had a treatment-related grade 2 infusion-related reaction. Potential TGF-β-related treatment-emergent skin adverse events occurred in 6 patients (7.2%; Table 3 and Supplementary Table SA.3); the lesions were well managed by surgical excision, which is the standard of care,^34^ and 1 patient received topical steroid treatment for keratoacanthoma and actinic keratosis. The safety profile was comparable between patients who had primary refractory disease and who had acquired resistance to prior anti-PD-(L)1 treatment (Supplementary Table SA.4).

**Table 3.** TRAEs occurring at any grade in ≥5% of patients or at grade ≥3 and any AEs of special interest.

	Any grade	Grade ≥3
TRAEs	61 (73.5)	19 (22.9)
Asthenia	23 (27.7)	3 (3.6)
Pruritus	19 (22.9)	2 (2.4)
Decreased appetite	14 (16.9)	1 (1.2)
Epistaxis	8 (9.6)	0
Fatigue	7 (8.4)	2 (2.4)
Arthralgia	6 (7.2)	1 (1.2)
Rash maculopapular	6 (7.2)	1 (1.2)
Diarrhea	6 (7.2)	0
Dry skin	5 (6.0)	0
Eczema	4 (4.8)	2 (2.4)
Anemia	4 (4.8)	1 (1.2)
Adrenal insufficiency	2 (2.4)	1 (1.2)
Rash macular	2 (2.4)	1 (1.2)
Vomiting	2 (2.4)	1 (1.2)
Squamous cell carcinoma of skin	2 (2.4)	1 (1.2)
Amylase increased	1 (1.2)	1 (1.2)^a^
Blood triglycerides increased	1 (1.2)	1 (1.2)
Bowen disease	1 (1.2)	1 (1.2)
Cataract	1 (1.2)	1 (1.2)
Folliculitis	1 (1.2)	1 (1.2)
General physical health deterioration	1 (1.2)	1 (1.2)
Hyponatremia	1 (1.2)	1 (1.2)
Leukocytosis	1 (1.2)	1 (1.2)
Lipase increased	1 (1.2)	1 (1.2)
Pemphigoid	1 (1.2)	1 (1.2)
Peripheral sensory neuropathy	1 (1.2)	1 (1.2)
Pneumonia	1 (1.2)	1 (1.2)^b^
AESIs		
Any TGF-β inhibition-mediated skin AE^c^	6 (7.2)	3 (3.6)
Any immune-related AE	6 (7.2)	2 (2.4)
Immune-related rash	6 (7.2)	2 (2.4)
Immune-related endocrinopathies: adrenal insufficiency	1 (1.2)	0

Data are *n* (%) in the safety set.

^a^Grade 4.

^b^Grade 5.

^c^Includes actinic keratosis, basal cell carcinoma, Bowen disease, hyperkeratosis, keratoacanthoma, lip squamous cell carcinoma, and squamous cell carcinoma of the skin MedDRA v21.0 preferred terms.

Abbreviations: AE, adverse event; AESI, adverse event of special interest; MedDRA, Medical Dictionary for Regulatory Activities; TGF-β, transforming growth factor beta; TRAE, treatment-related adverse event.

TGF-β1 gene expression in tumor samples at baseline did not predict response (Supplementary Fig. SA.4.A). RNAseq analysis suggested no relationship between response and tumor mutation count (Supplementary Fig. SA.4.B).

## Discussion

Of these 83 patients with heavily pretreated NSCLC, of whom three-quarters had received ≥3 prior therapies, 4 had a PR despite disease progression following anti-PD-(L)1 as their most recent treatment before bintrafusp alfa. The confirmed ORR as assessed by the IRC was 4.8% and did not exceed the 5% ORR threshold of the null hypothesis. The DCR was 18.1%, and 9.6% of patients had PFS ≥6 months. Notably, this study was conducted in a largely heterogenous population, with patients who had different tumor histologies, resistance types, PD-L1 expression levels, and last cancer therapies prior to bintrafusp alfa. While in a cohort of patients who were anti-PD-(L)1-naïve and received 2L or later bintrafusp alfa 1200 mg every 2 weeks, higher response rates were associated with PD-L1 expression, with an ORR of 37.0% in patients with PD-L1-positive tumors and 85.7% in those with PD-L1-high tumors compared with an ORR of 25.0% in all patients (PD-L1 evaluable and not evaluable), although the sample size was limited^28^; responses in this cohort of patients who had failure of prior anti-PD-(L)1 therapy were observed irrespective of PD-L1 expression (Fig. 3).

While resistance to checkpoint inhibitor therapy is not fully understood, possible underlying mechanisms include insufficient generation of antitumor T cells, inadequate function of tumor-specific T cells, or impaired formation of T-cell memory.^35^ TGF-β signaling in the local microenvironment could skew tumor-infiltrating T cells toward T regulatory phenotypes and attenuate the activation of CD8+ immune effector cells,^4,36^ and the TGF-β pathway signaling has been associated with resistance to checkpoint blockade.^23^ Therefore, inhibiting the TGF-β pathway might aid in overcoming anti-PD-(L)1 resistance. Of interest, all responses were observed in patients who had anti-PD-(L)1 therapy as last prior treatment and were observed in patients with primary refractory disease (ie, those patients who never had disease control with prior anti-PD-(L)1 therapy) and those who developed acquired resistance (ie, those patients who had temporary disease control with subsequent disease progression on prior anti-PD-(L)1 therapy; Fig. 1 and Supplementary Fig. SA.1). However, the modest clinical activity of bintrafusp alfa in this patient population suggests mechanisms of resistance to PD-(L)1 beyond TGF-β. TGF-β1 gene expression in tumor samples and the tumor mutation count showed no relationship with response. Exploratory biomarker analyses to determine differences between primary refractory vs acquired resistance have been performed, but no clear signals were observed in this small cohort. Further biomarker analysis is ongoing to identify potential mechanisms of resistance to anti-PD-(L)1 therapy in NSCLC and will be part of a separate manuscript.

Overall, bintrafusp alfa had a manageable safety profile in these patients, which was consistent with those of patients in the dose-escalation phase of this study^26^ and in the 2L cohort naïve to anti-PD-(L)1.^28^ Death from pneumonia, which was assessed as treatment-related by the investigator, occurred 8 days after the first dose in a patient who had an ongoing medical history of grade 3 pneumonia with parapneumonic pleural effusion 9 days before starting treatment with bintrafusp alfa. The safety profile was comparable between patients with primary refractory disease and acquired resistance to prior anti-PD-(L)1 treatment.

Study limitations include the lack of a comparator arm and the small number of patients, which make it difficult to interpret the magnitude of benefit for bintrafusp alfa. In addition, patients enrolled in this study had NSCLC that exhibited different anti-PD-(L)1-resistance types, histology, and PD-L1 expression levels and received a variety of prior treatments, reflecting a highly heterogeneous population. However, this patient population may more closely reflect the population of patients seen in real-world clinical practice. Trials of various treatment options have demonstrated limited efficacy for patients who are resistant or refractory to anti-PD-(L)1 treatment,^37-42^ and thus a strong need exists for novel treatment options for these difficult-to-treat patients.

In conclusion, although the study did not meet its primary endpoint, bintrafusp alfa showed clinical activity in some patients with heavily pretreated NSCLC who had primary refractory disease or who developed acquired resistance to prior treatment with anti-PD-(L)1 therapy, regardless of their prior treatment type or tumor PD-L1 expression levels.

## Supplementary Material

oyac253_suppl_Supplementary_Appendix

## Data Availability

Any requests for data by qualified scientific and medical researchers for legitimate research purposes will be subject to Merck’s Data Sharing Policy. All requests should be submitted in writing to Merck’s data sharing portal (https://www.merckgroup.com/en/research/our-approach-to-research-and-development/healthcare/clinical-trials/commitment-responsible-data-sharing.html). When Merck has a co-research, co-development, or co-marketing or co-promotion agreement, or when the product has been out-licensed, the responsibility for disclosure might be dependent on the agreement between parties. Under these circumstances, Merck will endeavor to gain agreement to share data in response to requests.

## References

[1] American Cancer Society. Cancer facts & figures 2022. Accessed March 25, 2022. https://www.cancer.org/content/dam/cancer-org/research/cancer-facts-and-statistics/annual-cancer-facts-and-figures/2022/2022-cancer-facts-and-figures.pdf

[2] Howlader N. Cancer statistics review, 1975-2014 - SEER statistics. Accessed May 27, 2022. https://seer.cancer.gov/archive/csr/1975_2014/results_merged/sect_15_lung_bronchus.pdf

[3] Altorki NK , Markowitz GJ, Gao D, et al. The lung microenvironment: an important regulator of tumour growth and metastasis. Nat Rev Cancer. 2019;19(1):9-31. 10.1038/s41568-018-0081-9. 30532012 PMC6749995

[4] Akhurst RJ , Hata A. Targeting the TGFβ signalling pathway in disease. Nat Rev Drug Discov. 2012;11(10):790-811. 10.1038/nrd3810. 23000686 PMC3520610

[5] Principe DR , Doll JA, Bauer J, et al. TGF-β: duality of function between tumor prevention and carcinogenesis. J Natl Cancer Inst. 2014;106(2):djt369. 10.1093/jnci/djt369. 24511106 PMC3952197

[6] Colak S , Ten Dijke P. Targeting TGF-β signaling in cancer. Trends Cancer. 2017;3(1):56-71. 10.1016/j.trecan.2016.11.008. 28718426

[7] Yang L , Pang Y, Moses HL. TGF-beta and immune cells: an important regulatory axis in the tumor microenvironment and progression. Trends Immunol. 2010;31(6):220-227. 10.1016/j.it.2010.04.002. 20538542 PMC2891151

[8] Zheng X , Turkowski K, Mora J, et al. Redirecting tumor-associated macrophages to become tumoricidal effectors as a novel strategy for cancer therapy. Oncotarget. 2017;8(29):48436-48452. 10.18632/oncotarget.17061. 28467800 PMC5564660

[9] Eser PÖ , Jänne PA. TGFβ pathway inhibition in the treatment of non-small cell lung cancer. Pharmacol Ther. 2018;184:112-130. 10.1016/j.pharmthera.2017.11.004. 29129643

[10] Meyers DE , Bryan PM, Banerji S, Morris DG. Targeting the PD-1/PD-L1 axis for the treatment of non-small-cell lung cancer. Curr Oncol. 2018;25(4):e324-e334. 10.3747/co.25.3976. 30111979 PMC6092051

[11] Gulley JL , Rajan A, Spigel DR, et al. Avelumab for patients with previously treated metastatic or recurrent non-small-cell lung cancer (JAVELIN solid tumor): dose-expansion cohort of a multicentre, open-label, phase 1b trial. Lancet Oncol. 2017;18(5):599-610. 10.1016/S1470-2045(17)30240-1. 28373005 PMC5522719

[12] Borghaei H , Paz-Ares L, Horn L, et al. Nivolumab versus docetaxel in advanced nonsquamous non-small-cell lung cancer. N Engl J Med. 2015;373(17):1627-1639. 10.1056/NEJMoa1507643. 26412456 PMC5705936

[13] Reck M , Rodriguez-Abreu D, Robinson AG, et al; KEYNOTE-024 Investigators. Pembrolizumab versus chemotherapy for PD-L1-positive non-small-cell lung cancer. N Engl J Med. 2016;375(19):1823-1833. 10.1056/NEJMoa1606774. 27718847

[14] Brahmer J , Reckamp KL, Baas P, et al. Nivolumab versus docetaxel in advanced squamous-cell non-small-cell lung cancer. N Engl J Med. 2015;373(2):123-135. 10.1056/NEJMoa1504627. 26028407 PMC4681400

[15] Rittmeyer A , Barlesi F, Waterkamp D, et al; OAK Study Group. Atezolizumab versus docetaxel in patients with previously treated non-small-cell lung cancer (OAK): a phase 3, open-label, multicentre randomised controlled trial. Lancet. 2017;389(10066):255-265. 10.1016/S0140-6736(16)32517-X. 27979383 PMC6886121

[16] Peters S , Gettinger S, Johnson ML, et al. Phase II trial of atezolizumab as first-line or subsequent therapy for patients with programmed death-ligand 1-selected advanced non-small-cell lung cancer (BIRCH). J Clin Oncol. 2017;35(24):2781-2789. 10.1200/JCO.2016.71.9476. 28609226 PMC5562171

[17] Peters S , Ramalingam SS, Paz-Ares L, et al. Nivolumab (NIVO) + low-dose ipilimumab (IPI) vs platinum-doublet chemotherapy (chemo) as first-line (1L) treatment (tx) for advanced non-small cell lung cancer (NSCLC): CheckMate 227 Part 1 final. Ann Oncol. 2019;30(suppl 5):v913-914. 10.1093/annonc/mdz394.075.

[18] Hellmann MD , Ciuleanu TE, Pluzanski A, et al. Nivolumab plus ipilimumab in lung cancer with a high tumor mutational burden. N Engl J Med. 2018;378:2093-2104. 10.1056/NEJMoa1801946. 29658845 PMC7193684

[19] Gandhi L , Rodriguez-Abreu D, Gadgeel S, et al; KEYNOTE-189 Investigators. Pembrolizumab plus chemotherapy in metastatic non-small-cell lung cancer. N Engl J Med. 2018;378:2078-2092. 10.1056/NEJMoa1801005. 29658856

[20] Paz-Ares L , Luft A, Vicente D, et al; KEYNOTE-407 Investigators. Pembrolizumab plus chemotherapy for squamous non-small-cell lung cancer. N Engl J Med. 2018;379(21):2040-2051. 10.1056/NEJMoa1810865. 30280635

[21] Socinski MA , Jotte RM, Cappuzzo F, et al; IMpower150 Study Group. Atezolizumab for first-line treatment of metastatic nonsquamous NSCLC. N Engl J Med. 2018;378:2288-2301. 10.1056/NEJMoa1716948. 29863955

[22] NCCN Clinical Practice Guidelines in Oncology. Non-small cell lung cancer. V3.2022. Accessed March 25, 2022. https://www.nccn.org/professionals/physician_gls/pdf/nscl.pdf

[23] Mariathasan S , Turley SJ, Nickles D, et al. TGFβ attenuates tumour response to PD-L1 blockade by contributing to exclusion of T cells. Nature. 2018;554:544-548. 10.1038/nature25501. 29443960 PMC6028240

[24] Tauriello DVF , Palomo-Ponce S, Stork D, et al. TGFbeta drives immune evasion in genetically reconstituted colon cancer metastasis. Nature. 2018;554:538-543. 10.1038/nature25492. 29443964

[25] Lan Y , Zhang D, Xu C, et al. Enhanced preclinical antitumor activity of M7824, a bifunctional fusion protein simultaneously targeting PD-L1 and TGF-β. Sci Transl Med. 2018;10:eaan5488. 10.1126/scitranslmed.aan5488. 29343622

[26] Strauss J , Heery CR, Schlom J, et al. Phase I trial of M7824 (MSB0011359C), a bifunctional fusion protein targeting PD-L1 and TGFβ, in advanced solid tumors. Clin Cancer Res. 2018;24:1287-1295. 10.1158/1078-0432.CCR-17-2653. 29298798 PMC7985967

[27] David JM , Dominguez C, McCampbell KK, et al. A novel bifunctional anti-PD-L1/TGF-β Trap fusion protein (M7824) efficiently reverts mesenchymalization of human lung cancer cells. Oncoimmunology. 2017;6:e1349589. 10.1080/2162402X.2017.1349589. 29123964 PMC5665067

[28] Paz-Ares L , Kim TM, Vicente D, et al. Bintrafusp alfa, a bifunctional fusion protein targeting TGF-β and PD-L1, in second-line treatment of patients with NSCLC: results from an expansion cohort of a phase 1 trial. J Thorac Oncol. 2020;15:1210-1222. 10.1016/j.jtho.2020.03.003. 32173464 PMC8210474

[29] Vugmeyster Y , Wilkins J, Harrison-Moench E, et al. Selection of the recommended phase 2 dose (RP2D) for M7824 (MSB0011359C), a bifunctional fusion protein targeting TGF-β and PD-L1. J Clin Oncol. 2018;36:2566-2566. 10.1200/jco.2018.36.15_suppl.2566.29945529

[30] Dobin A , Davis CA, Schlesinger F, et al. STAR: ultrafast universal RNA-seq aligner. Bioinformatics. 2013;29:15-21. 10.1093/bioinformatics/bts635. 23104886 PMC3530905

[31] Li H , Durbin R. Fast and accurate short read alignment with Burrows-Wheeler transform. Bioinformatics. 2009;25:1754-1760. 10.1093/bioinformatics/btp324. 19451168 PMC2705234

[32] Lai Z , Markovets A, Ahdesmaki M, et al. VarDict: a novel and versatile variant caller for next-generation sequencing in cancer research. Nucleic Acids Res. 2016;44:e108. 10.1093/nar/gkw227. 27060149 PMC4914105

[33] McLaren W , Gil L, Hunt SE, et al. The Ensembl Variant Effect Predictor. Genome Biol. 2016;17:122. 10.1186/s13059-016-0974-4. 27268795 PMC4893825

[34] Alain J. Management of keratoacanthoma. Curr Dermatol Rep. 2012;1:64-68.

[35] Jenkins RW , Barbie DA, Flaherty KT. Mechanisms of resistance to immune checkpoint inhibitors. Br J Cancer. 2018;118:9-16. 10.1038/bjc.2017.434. 29319049 PMC5765236

[36] Ahmadzadeh M , Rosenberg SA. TGF-beta 1 attenuates the acquisition and expression of effector function by tumor antigen-specific human memory CD8 T cells. J Immunol. 2005;174(9):5215-5223. 10.4049/jimmunol.174.9.5215. 15843517 PMC2562293

[37] Fong L , Forde PM, Powderly JD, et al. Safety and clinical activity of adenosine A2a receptor (A2aR) antagonist, CPI-444, in anti-PD1/PDL1 treatment-refractory renal cell (RCC) and non-small cell lung cancer (NSCLC) patients. J Clin Oncol. 2017;35:3004-3004. 10.1200/jco.2017.35.15_suppl.3004.

[38] Leger PD , Rothschild S, Castellanos E, et al. Response to salvage chemotherapy following exposure to immune checkpoint inhibitors in patients with non-small cell lung cancer. J Clin Oncol. 2017;35:9084-9084. 10.1200/jco.2017.35.15_suppl.9084.

[39] Schvartsman G , Peng SA, Bis G, et al. Response rates to single-agent chemotherapy after exposure to immune checkpoint inhibitors in advanced non-small cell lung cancer. Lung Cancer. 2017;112:90-95. 10.1016/j.lungcan.2017.07.034. 29191606

[40] Schoenfeld JD , Giobbie-Hurder A, Ranasinghe S, et al. Durvalumab plus tremelimumab alone or in combination with low-dose or hypofractionated radiotherapy in metastatic non-small-cell lung cancer refractory to previous PD(L)-1 therapy: an open-label, multicentre, randomised, phase 2 trial. Lancet Oncol. 2022;23(2):279-291. 10.1016/S1470-2045(21)00658-6. 35033226 PMC8813905

[41] Niu J , Maurice-Dror C, Lee DH, et al. First-in-human phase 1 study of the anti-TIGIT antibody vibostolimab as monotherapy or with pembrolizumab for advanced solid tumors, including non-small-cell lung cancer. Ann Oncol. 2022;33(2):169-180. 10.1016/j.annonc.2021.11.002. 34800678

[42] Hellmann MD , Jänne PA, Opyrchal M, et al. Entinostat plus pembrolizumab in patients with metastatic NSCLC previously treated with anti-PD-(L)1 therapy. Clin Cancer Res. 2021;27(4):1019-1028. 10.1158/1078-0432.CCR-20-3305. 33203644 PMC7887114

