# Supplementary material for: Bintrafusp Alfa, a Bifunctional Fusion Protein Targeting TGF-β and PD-L1, in Patients with Non-Small Cell Lung Cancer Resistant or Refractory to Immune Checkpoint Inhibitors
Source: Oncologist. 2022 Dec 26;28(3):258–67. doi: 10.1093/oncolo/oyac253 (PMC10020814; doi:10.1093/oncolo/oyac253)
Supplement: oyac253_suppl_Supplementary_Appendix [file oyac253_suppl_supplementary_appendix.docx]

**Appendix**

## Table A.1. Investigator Sites

| **Site number** | **Principal investigator** | **Site/institution** | **Total**  **(N=83)** |
| --- | --- | --- | --- |
| 509 | Fabrice Barlesi | Aix Marseille University, Hôpital de la Timone, Marseille, France | 14 |
| 554 | Nicolas Isambert | Centre Georges François Leclerc, Dijon, France | 8 |
| 546 | Enriqueta Felip Font | Hospital Universitari Vall d'Hebron, Barcelona, Spain | 6 |
| 114 | Julio Peguero | Oncology Consultants PA, Houston, TX, USA | 4 |
| 805 | Byoung Chul Cho | Severance Hospital, Yonsei University, Seoul, Republic of Korea | 4 |
| 806 | Dae Ho Lee | Asian Medical Center, Seoul, Republic of Korea | 4 |
| 504 | Guy Jerusalem | CHU Sart Tilman, Liege, Belgium | 3 |
| 508 | Nicolas Penel | Department of Medical Oncology, Centre Oscar Lambret and Lille University Hospital, Lille, France | 3 |
| 514 | Esma Saada-Bouzid | Centre Antoine Lacassagne, Service d'Hématologie Oncologie, Nice, France | 3 |
| 524 | Federico Longo Muñoz | Servicio de Oncologia, Hospital Universitario Ramon y Cajal, Madrid, Spain | 3 |
| 106 | Minal Barve | Mary Crowley Cancer Research Centers, Dallas, TX, USA | 2 |
| 131 | Donald Richards | Texas Oncology, P.A. – Tyler, Tyler, TX, USA | 2 |
| 143 | Karen Kelly | University of California Davis Health System, San Francisco, CA, USA | 2 |
| 149 | Howard Safran | Rhode Island Hospital, Providence, RI, USA | 2 |
| 501 | Jean-Luc Canon | Grand Hôpital de Charleroi, Charleroi, Belgium | 2 |
| 512 | Carole Gourmelon | ICO - Site René Gauducheau, Saint-Herblain, France | 2 |
| 526 | Massimo Di Nicola | Fondazione IRCCS Istituto Nazionale dei Tumori, Milano, Italy | 2 |
| 541 | Oscar Jose Juan Vidal | Hospital Universitari i Politecnic La Fe, Valencia, Spain | 2 |
| 614 | Craig Gedye | Calvary Mater Newcastle, Waratah, NSW, Australia | 2 |
| 103 | Aung Naing | University of Texas MD Anderson Cancer Center – Investigational Cancer Therapeutics, Houston, TX, USA | 1 |
| 104 | Michael Wertheim | Hematology – Oncology Associates of the Treasure Coast, Port St. Lucie, FL, USA | 1 |
| 112 | John Morris | UC Health, LLC, Cincinnati, OH, USA | 1 |
| 119 | Patricia Rich | Southeastern Regional Medical Center, Newman, GA, USA | 1 |
| 120 | Petros Nikolinakos | University Cancer & Blood Center, Athens, GA, USA | 1 |
| 121 | Jayne Gurtler | Metairie Oncologists, LLC, Metairie, LA, USA | 1 |
| 128 | Allen Cohn | Rocky Mountain Cancer Centers, LLP, Denver, CO, USA | 1 |
| 531 | Maria Jose Flor Oncala | Hospital Universitario Virgen del Rocio, Sevilla, Spain | 1 |
| 544 | Jean-Pascal Machiels | Cliniques Universitaires Saint-Luc, Bruxelles, Belgium | 1 |
| 604 | Gary Richardson | Cabrini Hospital Malvern, Malvern, Victoria, Australia | 1 |
| 605 | Rachel Roberts-Thomson | The Queen Elizabeth Hospital, Woodville South, South Australia, Australia | 1 |
| 611 | Chee Lee | St. George Hospital, Kogarah, NSW, Australia | 1 |
| 807 | Ki Hyeong Lee | Chungbuk National University Hospital, Cheongju-si, Republic of Korea | 1 |

## Table A.2. Clinical Response to Bintrafusp Alfa as Assessed by the Investigators

|  | **Primary refractory**  **(n=35)** | **Acquired resistance**  **(n=45)** | **Total**  **(N=83)^a^** |
| --- | --- | --- | --- |
| Best overall response  Complete response  Partial response  Stable disease  Progressive disease  Not evaluable | 0  3 (8.6)  3 (8.6)  22 (62.9)  7 (20) | 0  1 (2.2)  14 (31.1)  22 (48.9)  8 (17.8) | 0  4 (4.8)  17 (20.5)  46 (55.4)  16 (19.3) |
| Objective response rate | 3 (8.6; 1.8-23.1) | 1 (2.2; 0.1-11.8) | 4 (4.8; 1.3-11.9) |
| Disease control rate | 6 (17.1; 6.6-33.6) | 15 (33.3; 20.0-49.0) | 21 (25.3; 16.4-36.0) |

Data are n (%) or n (%; 95% CI) according to investigator assessments per Response Evaluation Criteria in Solid Tumors version 1.1.

^a^ Primary refractory or acquired resistance status is not available for 3 of the 83 patients.

**Table A.3.** **Patients Reporting Adverse Events of Special Interest**

|  | **Any grade**  **n (%)** | **Grade 3**  **n (%)** |
| --- | --- | --- |
| Any immune-related adverse event  Immune-related rash  Pruritus  Pemphigoid  Rash macular  Dermatitis acneiform  Rash  Rash erythematous  Rash papular  Immune-related endocrinopathies: adrenal insufficiency  Adrenal insufficiency  Any TGF-β inhibition–mediated skin adverse event^a^  Keratoacanthoma  Squamous cell carcinoma of skin  Actinic keratosis  Bowen disease  Basal cell carcinoma | 6 (7.2)  6 (7.2)  2 (2.4)  1 (1.2)  1 (1.2)  1 (1.2)  1 (1.2)  1 (1.2)  1 (1.2)  1 (1.2)  1 (1.2)  6 (7.2)  4 (4.8)  3 (3.6)  3 (3.6)  1 (1.2)  1 (1.2) | 2 (2.4)  2 (2.4)  0  1 (1.2)  1 (1.2)  0  0  0  0  0  0  3 (3.6)  0  2 (2.4)  0  1 (1.2)  0 |

MedDRA, Medical Dictionary for Regulatory Activities; TGF-β, transforming growth factor beta.

Data are n (%) in the safety set.

^a^ Includes actinic keratosis, basal cell carcinoma, Bowen disease, hyperkeratosis, keratoacanthoma, lip squamous cell carcinoma, and squamous cell carcinoma of the skin MedDRA v21.0 preferred terms.

## Table A.4. Patients Reporting TRAEs Occurring at Any Grade in ≥5% of Patients or at Grade ≥3 by Subgroup

|  | **Primary refractory  (n=35)** | | **Acquired resistance  (n=45)** | |
| --- | --- | --- | --- | --- |
|  | **Any grade** | **Grade ≥3** | **Any grade** | **Grade ≥3** |
| Any TRAE, n (%)  Asthenia  Decreased appetite  Pruritus  Epistaxis  Anemia  Fatigue  Gingival bleeding  Keratoacanthoma  Arthralgia  Rash macular  Vomiting  Dysgeusia  Myalgia  Skin lesion  Telangiectasia  Weight decreased  Adrenal insufficiency  Blood triglycerides increased  Bowen disease  Cataract  Eczema  General physical health deterioration  Hyponatremia  Pemphigoid  Peripheral sensory neuropathy  Pneumonia  Squamous cell carcinoma of skin  Diarrhea  Dry skin  Rash maculopapular  Rash  Amylase increased  Folliculitis  Leukocytosis  Lipase increased | 26 (74.3)  12 (34.3)  10 (28.6)  5 (14.3)  5 (14.3)  4 (11.4)  3 (8.6)  3 (8.6)  3 (8.6)  2 (5.7)  2 (5.7)  2 (5.7)  2 (5.7)  2 (5.7)  2 (5.7)  2 (5.7)  2 (5.7)  1 (2.9)  1 (2.9)  1 (2.9)  1 (2.9)  1 (2.9)  1 (2.9)  1 (2.9)  1 (2.9)  1 (2.9)  1 (2.9)  1 (2.9)  1 (2.9)  1 (2.9)  0  0  0  0  0  0 | 13 (37.1)  3 (8.6)  1 (2.9)  1 (2.9)  0  1 (2.9)  1 (2.9)  0  0  1 (2.9)  1 (2.9)  1 (2.9)  0  0  0  0  0  1 (2.9)  1 (2.9)  1 (2.9)  1 (2.9)  1 (2.9)  1 (2.9)  1 (2.9)  1 (2.9)  1 (2.9)  1 (2.9)^a^  1 (2.9)  0  0  0  0  0  0  0  0 | 33 (73.3)  9 (20.0)  4 (8.9)  13 (28.9)  3 (6.7)  0  4 (8.9)  1 (2.2)  1 (2.2)  4 (8.9)  0  0  0  2 (4.4)  0  0  1 (2.2)  1 (2.2)  0  0  0  3 (6.7)  0  0  0  0  0  1 (2.2)  5 (11.1)  4 (8.9)  6 (13.3)  3 (6.7)  1 (2.2)  1 (2.2)  1 (2.2)  1 (2.2) | 6 (13.3)  0  0  1 (2.2)  0  0  1 (2.2)  0  0  0  0  0  0  0  0  0  0  0  0  0  0  1 (2.2)  0  0  0  0  0  0  0  0  1 (2.2)  0  1 (2.2)^b^  1 (2.2)  1 (2.2)  1 (2.2) |

TRAE, treatment-related adverse event.

^a^ Grade 5.

^b^ Grade 4.

## Figure A.1. Best response to bintrafusp alfa. Best percentage change in target lesions from baseline as assessed by the IRC, according to PD-(L)1 resistance status (A) or by previous anticancer therapy (B). Dashed lines at 20% and –30% indicate thresholds for progressive disease and partial response, respectively.


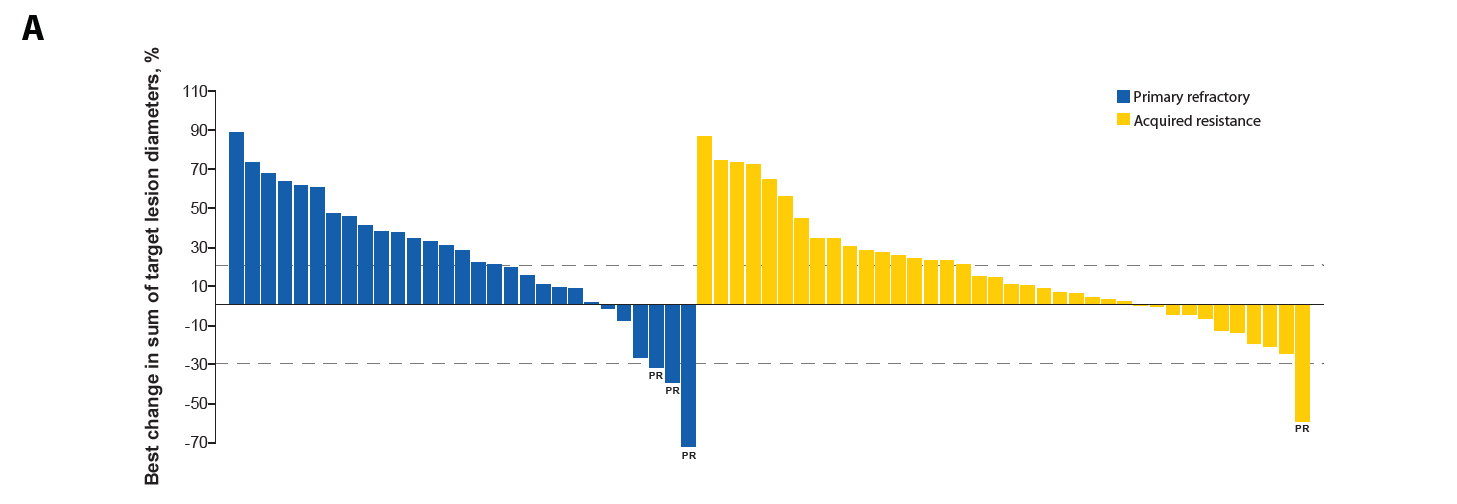


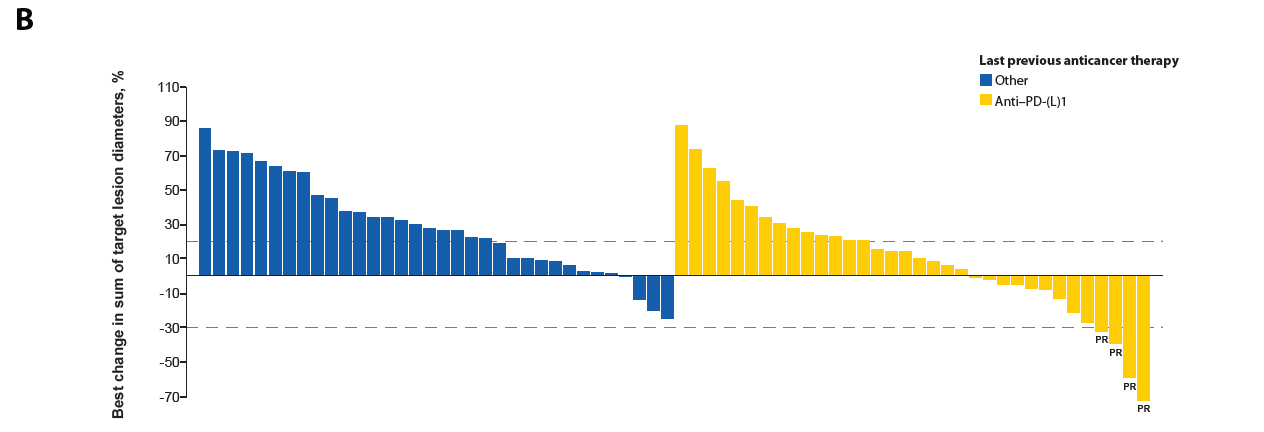


IRC, independent review committee; PD-L1, programmed cell death 1 ligand 1.

## Figure A.2. Kaplan-Meier curve of PFS according to RECIST 1.1 as adjudicated by the IRC.


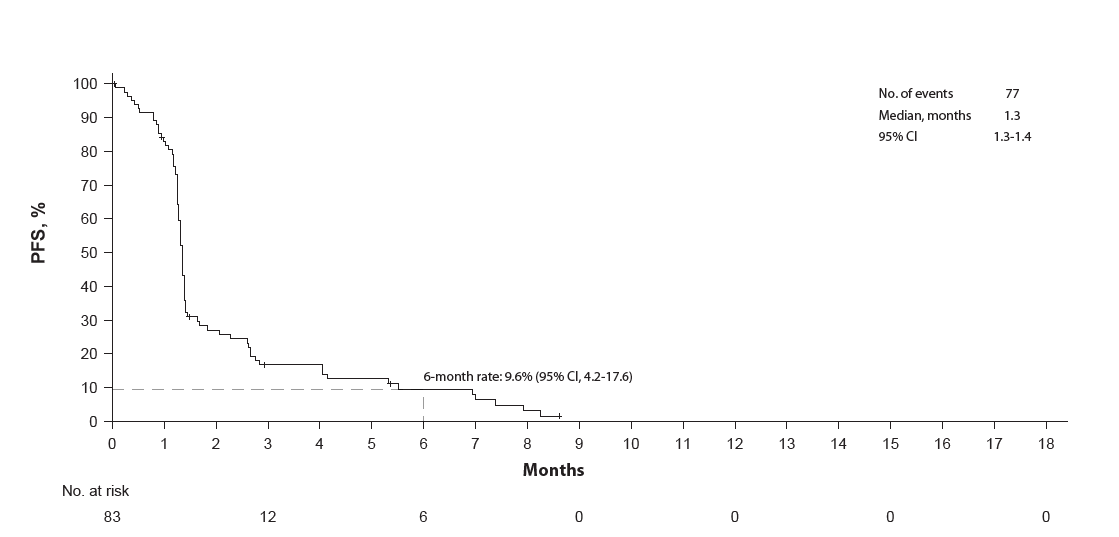


IRC, independent review committee; PFS, progression-free survival; RECIST 1.1, Response Evaluation Criteria in Solid Tumors version 1.1.

## Figure A.3. Kaplan-Meier curve of OS.


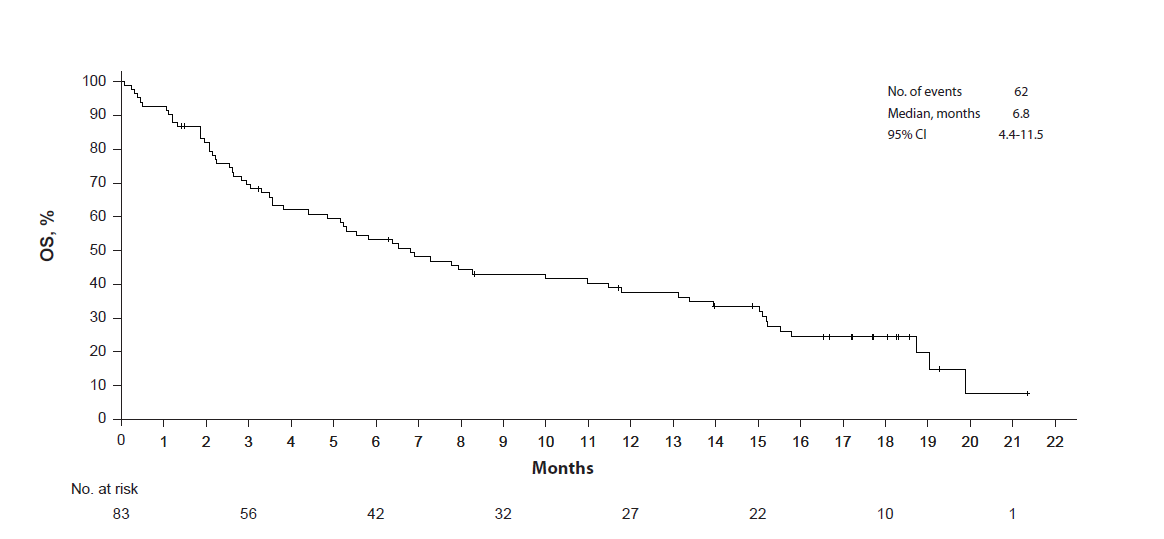


OS, overall survival.

## Figure A.4. Tumor cell *TGFB1* gene expression patterns (A) and tumor mutation count (B) as predictors of response.


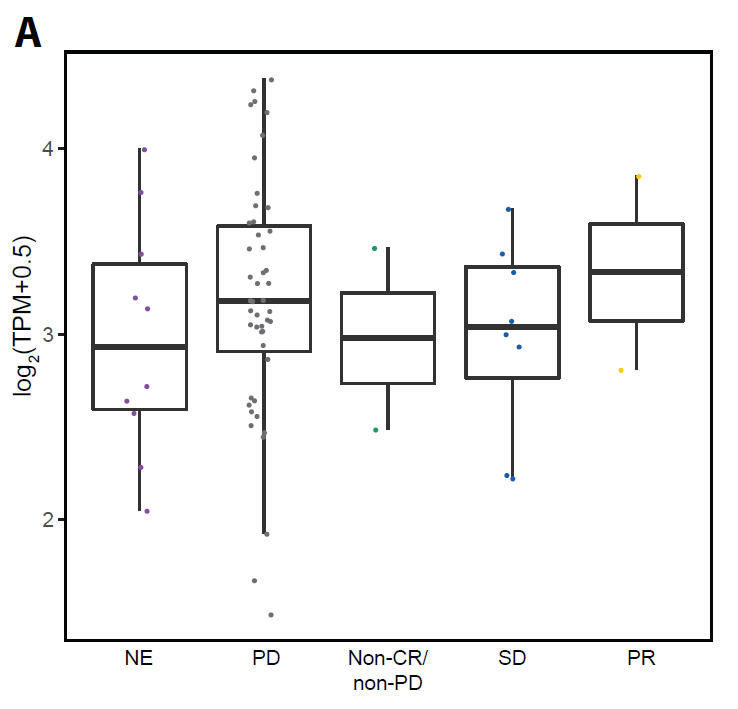


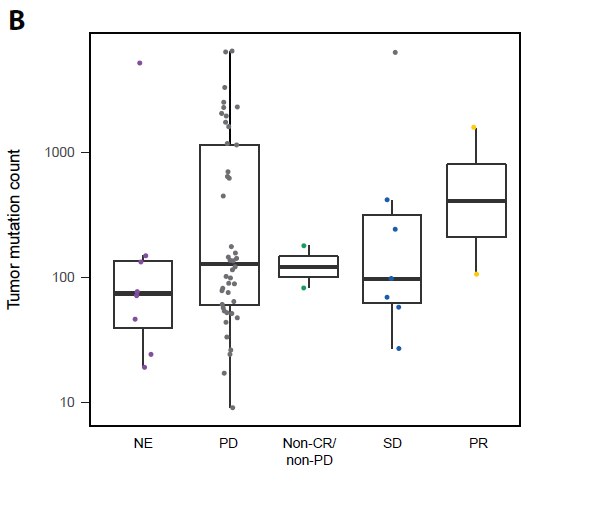


Confirmed best overall response (CBOR) per IRC assessments are shown. For the *TGFB1* expression analysis, 14 patients were excluded due to the absence of an RNA sample (CBOR of PR [n=1], SD [n=1], PD [n=10], and NE [n=2]), and an additional 5 patients were excluded due to RNAseq quality control failure (CBOR of PR [n=1], SD [n=1], PD [n=1], and NE [n=2]). For the tumor mutation count analysis, 19 patients were excluded due to lack of paired RNA and DNA samples (CBOR of PR [n=1], SD [n=2], PD [n=12], and NE [n=4]).

CR, complete response; IRC, independent review committee; NE, not evaluable; PD, progressive disease; PR, partial response; SD, stable disease; TPM, transcript per million.
